# Cost of community-led larval source management and house improvement for malaria control: a cost analysis within a cluster-randomized trial in a rural district in Malawi

**DOI:** 10.1186/s12936-021-03800-4

**Published:** 2021-06-13

**Authors:** Mphatso Dennis Phiri, Robert S. McCann, Alinune Nathanael Kabaghe, Henk van den Berg, Tumaini Malenga, Steven Gowelo, Tinashe Tizifa, Willem Takken, Michèle van Vugt, Kamija S. Phiri, Dianne J. Terlouw, Eve Worrall

**Affiliations:** 1 Malaria Epidemiology Group, Malawi-Liverpool-Wellcome Trust Clinical Research Programme, Blantyre, Malawi; 2 Laboratory of Entomology, Wageningen University & Research, Wageningen, The Netherlands; 3 School of Public Health and Family Medicine, College of Medicine, University of Malawi, Blantyre, Malawi; 4 Center for Vaccine Development and Global Health, University of Maryland School of Medicine, Baltimore, USA; 5 Center for Tropical Medicine and Travel Medicine, Department of Infectious Diseases, Division of Internal Medicine, Amsterdam-UMC, Location AMC, University of Amsterdam, Amsterdam, The Netherlands; 6 Liverpool School of Tropical Medicine, Liverpool, UK

**Keywords:** Malaria, Cost analysis, House improvement, Larval source management, Community-led

## Abstract

**Background:**

House improvement (HI) to prevent mosquito house entry, and larval source management (LSM) targeting aquatic mosquito stages to prevent development into adult forms, are promising complementary interventions to current malaria vector control strategies. Lack of evidence on costs and cost-effectiveness of community-led implementation of HI and LSM has hindered wide-scale adoption. This study presents an incremental cost analysis of community-led implementation of HI and LSM, in a cluster-randomized, factorial design trial, in addition to standard national malaria control interventions in a rural area (25,000 people), in southern Malawi.

**Methods:**

In the trial, LSM comprised draining, filling, and *Bacillus thuringiensis israelensis-*based larviciding, while house improvement (henceforth HI) involved closing of eaves and gaps on walls, screening windows/ventilation spaces with wire mesh, and doorway modifications. Communities implemented all interventions. Costs were estimated retrospectively using the ‘ingredients approach’, combining ‘bottom-up’ and ‘top-down approaches’, from the societal perspective. To estimate the cost of independently implementing each intervention arm, resources shared between trial arms (e.g. overheads) were allocated to each consuming arm using proxies developed based on share of resource input quantities consumed. Incremental implementation costs (in 2017 US$) are presented for HI-only, LSM-only and HI + LSM arms. In sensitivity analyses, the effect of varying costs of important inputs on estimated costs was explored.

**Results:**

The total economic programme costs of community-led HI and LSM implementation was $626,152. Incremental economic implementation costs of HI, LSM and HI + LSM were estimated as $27.04, $25.06 and $33.44, per person per year, respectively. Project staff, transport and labour costs, but not larvicide or screening material, were the major cost drivers across all interventions. Costs were sensitive to changes in staff costs and population covered.

**Conclusions:**

In the trial, the incremental economic costs of community-led HI and LSM implementation were high compared to previous house improvement and LSM studies. Several factors, including intervention design, year-round LSM implementation and low human population density could explain the high costs. The factorial trial design necessitated use of proxies to allocate costs shared between trial arms, which limits generalizability where different designs are used. Nevertheless, costs may inform planners of similar intervention packages where cost-effectiveness is known.

*Trial registration* Not applicable. The original trial was registered with The Pan African Clinical Trials Registry on 3 March 2016, trial number PACTR201604001501493

**Supplementary Information:**

The online version contains supplementary material available at 10.1186/s12936-021-03800-4.

## Background

Despite significant reductions in cases and deaths between 2000 and 2015, *Plasmodium falciparum* malaria remains an important global health problem, especially in Africa. This reduction was largely attributed to vector control interventions: insecticide-treated nets (ITN) and indoor residual spraying (IRS) [1, 2]. In addition to ITNs and IRS, intermittent preventative therapy in pregnancy with sulfadoxine–pyrimethamine (IPTp), prompt diagnosis and effective case management with rapid diagnostic tests (RDTs) and artemisinin-based combination therapy (ACT) have been key to the recent reduction in burden [2]. However, resistance to insecticides used for ITN and IRS and other challenges have threatened this success: in 2017, 231 million cases were reported compared to 214 million in 2015 [3, 4]. In addition to insecticide resistance, outdoor mosquito resting and biting behaviour pose a challenge [5]. Use of interventions with different modes of action that target both aquatic and adult mosquito stages could mitigate against these challenges and ensure progress towards current control and elimination targets is sustained [5–8].

Larval source management (LSM) and house improvement (HI) are existing methods of vector control with different modes of action to ITNs and IRS. Both LSM and HI have been shown to reduce malaria transmission and morbidity and, therefore, could be used as complementary interventions by malaria control programmes [9–12], likely increasing programme impact but also increasing costs and thus requiring careful consideration.

LSM includes any or all of habitat modification, habitat manipulation, biological control, or larviciding; targets aquatic mosquito stages to prevent development into adult forms; and has been shown to reduce malaria transmission and morbidity [9, 13]. The World Health Organization (WHO) recommends larviciding in areas where larval sources are “few, fixed and findable” [13]. LSM is a potential complementary option for malaria control in addition to long-lasting ITNs (LLINs) and IRS. Despite LSM’s effectiveness, perceptions of high costs, logistical challenges and lack of evidence on cost and cost-effectiveness hinder large-scale implementation [14, 15]. Notably, previous studies on LSM focused on bacterial larviciding, rarely including draining and filling, the two other components of LSM [13]. Moreover, in rural areas with moderate to high malaria transmission there is a gap in evidence on the costs and effectiveness of LSM [9, 10, 13].

Similarly, despite historical success and promise for malaria control, structural house improvement interventions, which prevent mosquito house entry, are not prioritized in national malaria control policies, although they are increasingly gaining prominence in global socioeconomic development agenda [16]. Improved housing, ranging from structural house modifications aimed at reducing mosquito house entry, to modern housing designs, is associated with reduced risk of malaria infection, albeit to varying magnitudes [17–19]. A recent systematic review that included six cluster randomised controlled trials of structural house modifications in Africa, only three of which have been published to date, concluded that house screening reduces malaria infection and transmission [20]. However, apart from effectiveness, very few studies reported costs of house improvement interventions [12, 19, 21]. Importantly, these studies included various components of house improvement, from structural modifications/improvements to fitting of insecticide delivery devices (e.g. eave tubes) to building new/‘modern’ houses. No study has included implementation costs of a community-led house improvement or larval source management intervention.

This study presents a trial-based cost analysis, without comparison of effectiveness outcomes [22], conducted to estimate the incremental costs of implementing community-led HI and LSM, alone or in combination, in addition to standard national malaria control programme (NMCP) interventions. Since the purpose was to estimate the incremental cost of adding these new interventions, including the community engagement programme, to standard practice, the costs of existing NMCP interventions were not collected or analysed; these interventions are already recognised as being cost-effective. From a societal perspective, the following costs were estimated: incremental total and per capita programme financial and economic costs of implementing each intervention arm in the Majete Malaria Project larval source management and house improvement trial conducted in Chikwawa district, southern Malawi [23].

## Methods

### Study area

**Fig. 1:**
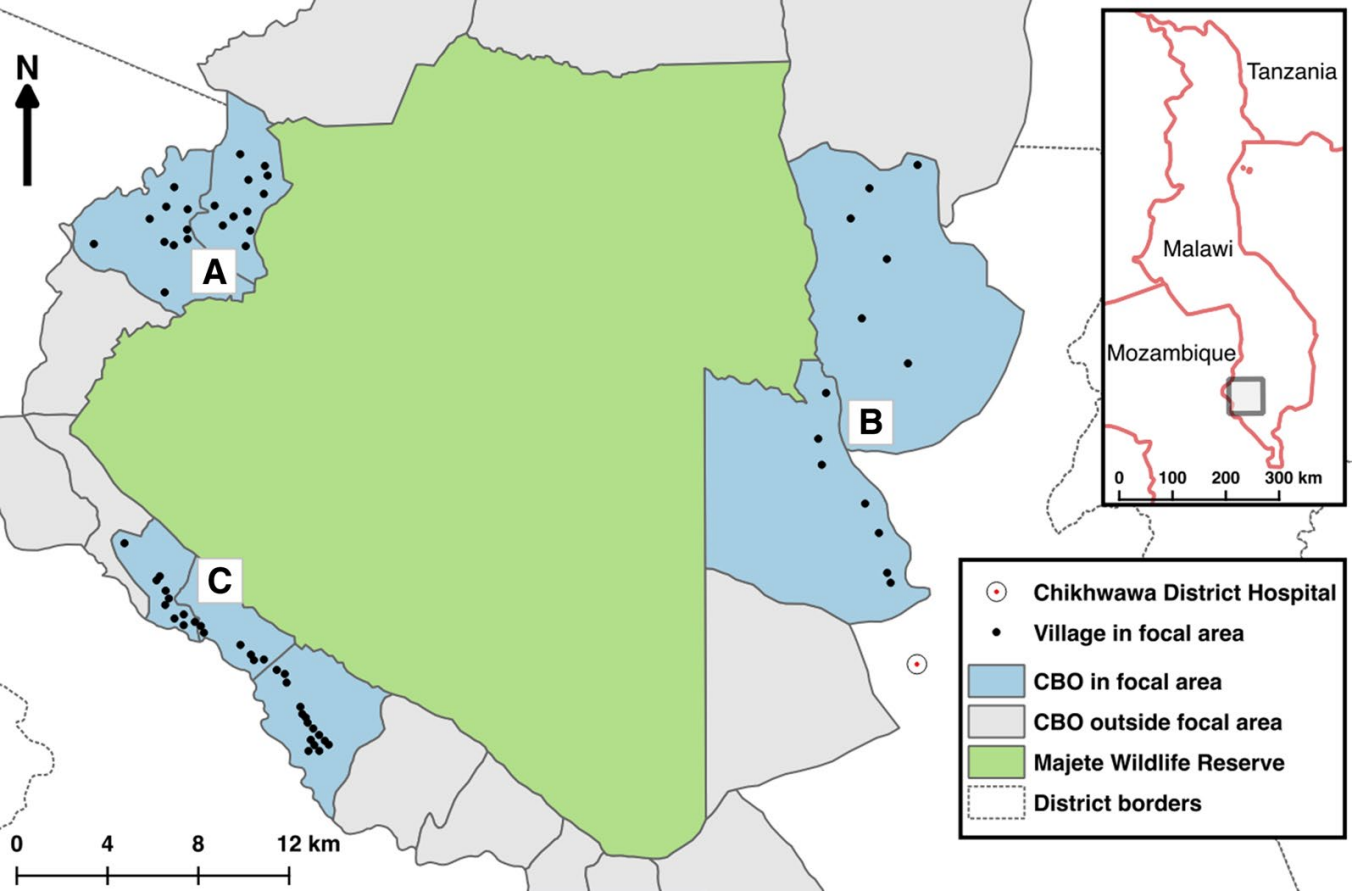
Map of Majete Wildlife Reserve, showing the Majete Malaria Project (MMP) study site. A, B and C denote ‘focal areas’ in which the trial was implemented. Focal areas were a group a villages selected to coincide with The Hunger Project epicentres—pre-existing organizational units of socio economic development and community engagement—within which the MMP was implemented. The main project field station from which field staff operated and coordinated field activities (including planning and oversight, storage of project items) was located close to Focal area B. (Adapted with permission from Kabaghe et al. [46])

The Majete Malaria Project larval source management and house improvement trial, hereafter ‘MMP LSM/HI trial’, was part of an operational malaria control project, the Majete Malaria Project (MMP) implemented from 2014 to 2019. MMP was a collaboration between academic institutions, non-governmental organizations (African Parks-Majete (AP) and The Hunger Project (THP)) and the Malawi Government Ministry of Health and Chikwawa District Health Office (DHO). The study site and design are reported in detail elsewhere [23]. Briefly, the Majete Wildlife Reserve (MWR) is a wildlife conservation area in Chikwawa district, about 60 kms south of Blantyre city, in Malawi. The MMP study area covered an estimated 260 km^2^, which included three ‘focal areas’ surrounding the MWR (Fig. 1). In 2015, the MMP study area catchment population was estimated at 25,000 people. From April 2015 to April 2016, malaria parasite prevalence in children aged 6–59 months was 33.8% (95% confidence interval 30.8–36.9%) [24]. The main source of income for the communities surrounding MWR is subsistence farming [25].

In Malawi, malaria transmission is perennial, peaking roughly with the rainy season from November to April [26]. The main malaria vectors are *Anopheles gambiae *sensu stricto (s.s.), *Anopheles arabiensis* and *Anopheles funestus* [27]. Malaria control relies on long-lasting ITNs (LLINs), IPTp, prompt diagnosis and effective case management with RDTs and ACT (artemether–lumefantrine), respectively. At the time of the study, IRS was being implemented in selected districts, which did not include Chikwawa district. Both LSM and house screening were used for malaria control in the 1900s, but only LSM, specifically in targeted communities, is included in the 2017–2022 NMCP strategic plan, although it is yet to be implemented to date [23, 26].

The main objective of MMP was to reduce malaria transmission through implementation of community-led interventions including LSM and house improvement, in addition to standard NMCP interventions [23]. Community engagement, with a view towards sustainability, was a core objective of the project [15–18]. Community volunteers, called *health animators*, facilitated intensive community engagement workshops throughout the MMP catchment area as well as LSM and house improvement intervention implementation by community members in their respective villages [23, 28]. Health animators facilitated the ‘epicentre approach’ initiated and managed by THP in communities around MWR. An epicentre is a grouping of villages through which communities are supported to create sustainable solutions for their own socio-economic problems, with a view towards sustainable self-reliance [29]. The MMP was embedded into this epicentre approach with particular focus on sustainable malaria control as an additional means towards socio-economic development [23].

### Trial design

The MMP LSM/HI trial used a cluster-randomized 2 × 2 factorial design to assess the effect of LSM and HI on malaria transmission when added to standard national malaria control programme (NMCP) interventions at scale-up for impact targets, over a 2-year period. The village was the unit of intervention implementation, so that all households in each village were targeted to receive the trial intervention, as per the cluster’s treatment arm [24]. The four trial arms were: (1) NMCP + HI; (2) NMCP + LSM; (3) NMCP + HI + LSM; (4) NMCP only (control arm); hereinafter HI, LSM, HI + LSM and control arms, respectively. NMCP interventions comprised LLINs; IPTp; and prompt diagnosis and effective case management with RDTs and ACT, respectively. In addition, as part of the community engagement programme led by THP, all trial arms implemented ‘malaria village workshops’ aimed at increasing awareness and uptake of the NMCP interventions [28]. Each workshop, attended by residents from one or more villages and facilitated by a health animator, involved discussing a malaria-related topic. These workshops started in all MMP catchment area villages 1 year before the LSM/HI trial and continued throughout the trial period. Additional workshops in HI and/or LSM villages focused on HI and/or LSM, as per trial arm [25]. No IRS was implemented in the study area during the trial period [23]. The MMP was not responsible for implementation of NMCP interventions; rather, these interventions were implemented by the Chikwawa DHO as done throughout the district [24].

### Trial interventions

MMP project management and technical staff from the academic institutions provided oversight and coordinated logistics relating to intervention implementation. THP and AP facilitated community engagement and intervention uptake. The district health office granted permission and facilitated integration of trial interventions into the existing health system structures. Detailed staff roles are included in Additional file 1. Broadly, implementation of trial interventions followed a step-wise approach, starting with seeking community leaders’ approval. Following discussions with village leaders and trainings, community members implemented interventions, as below.

**Fig. 2:**
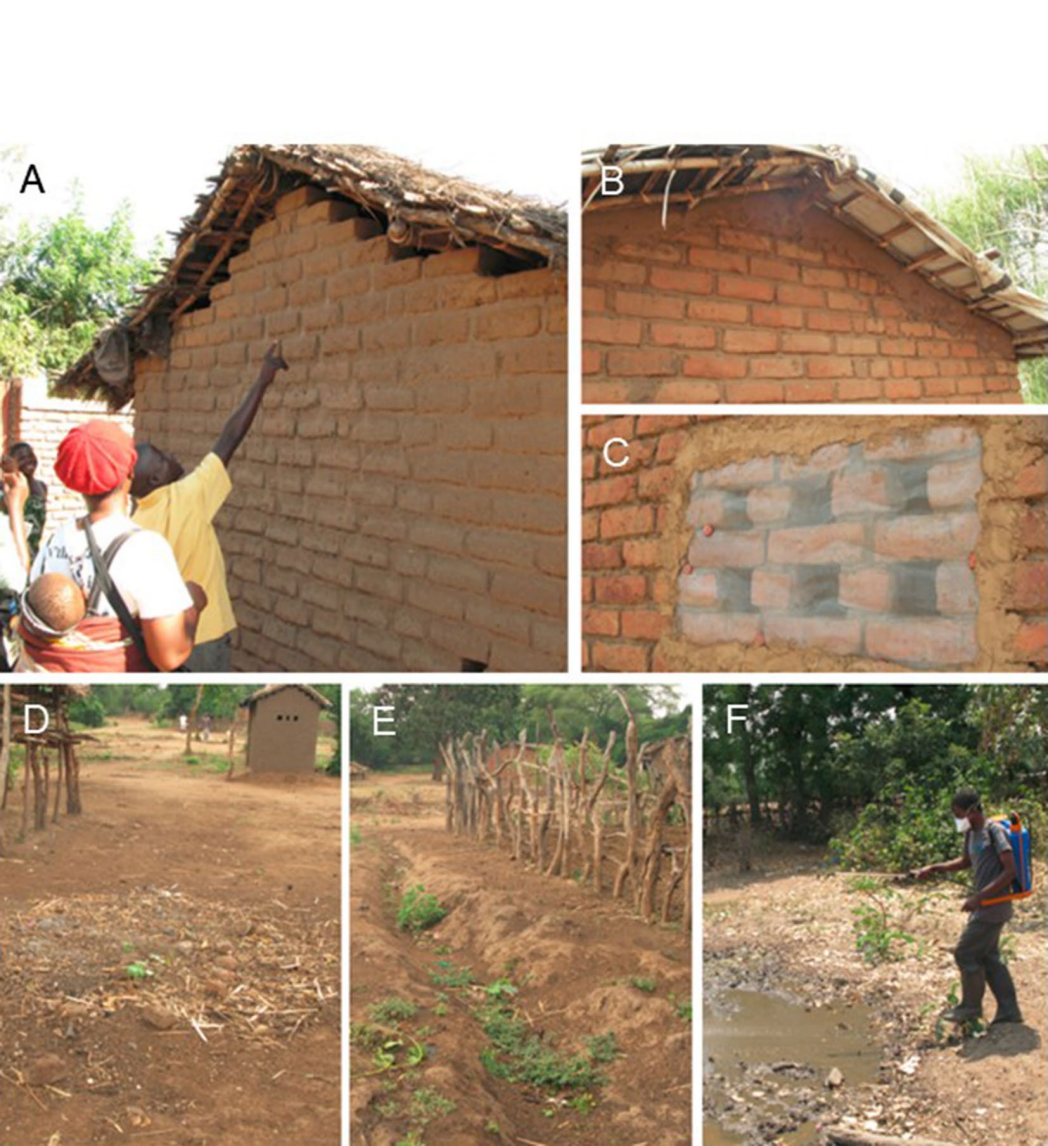
House improvement (top panels) and larval source management (bottom panels). **A** Pre-intervention; **B** closed gable; **C** screened ‘windows’; **D** former water body filled with soil; **E** drainage passage created to prevent standing water; **F** trained LSM committee member applying *Bacillus* *thuringiensis* israelensis to water bodies. (Reprinted with permission from van den Berg et al. [28])

LSM consisted of draining, filling and bacterial larviciding (using *Bacillus thuringiensis israelensis*, abbreviated *Bti*, AM625 strain, commercial name: VectoBac WDG [Valent Biosciences, Libertyville IL, USA]) of standing water bodies. Following an initial training of trainers, health animators were trained on LSM rationale and methods and cascaded training to other community members. At the community level, village members first manually identified all water bodies on the ground, and produced hand-drawn maps, using their knowledge of their community topography. While satellite images were used in research sub-studies within the larger MMP project, no satellite images or other technology was used in identification and/or mapping of water bodies related to the community-led trial implementation. Using a predefined algorithm, community members targeted mapped water bodies for draining or filling, or larviciding if draining/filling were deemed inappropriate, e.g. for water bodies used for domestic purposes. Application of *Bti* was introduced later after communities had already started implementing draining and filling [28]. An LSM committee (up to 12 people per one or two villages) was chosen to oversee LSM, including the organization of draining and filling activities. The LSM committees were also directly responsible for *Bti* application. LSM activities occurred all year round: LSM-specific malaria workshops once every 2 weeks; *Bti* application weekly; the majority of draining and filling was conducted once off initially, with maintenance to prevent standing water as needed thereafter [28]. *Bti*, protective clothing (face masks and rubber boots) and spraying equipment were provided by the project but the communities conducted all mapping of water bodies, draining, filling and spraying, as well as pre- and post-spray larval sampling as a method for communities to monitor the programme (see Fig. 2). Apart from *Bti*, imported from the USA, all materials required were locally acquired.

House improvement entailed structural modification of houses to prevent mosquito entry, specifically closing of eaves, other walls gaps, and doorway modifications using locally available materials originally used in constructing the houses (mostly brick and mud) and screening holes/spaces used for ventilation, including windows, with wire mesh. Except for the wire mesh; heavy-duty scissors for cutting wire mesh; and measuring tape, all other materials, e.g. nails, brick and mud for closing eaves, wall gaps and door frame modifications for implementing HI were provided by communities (see Fig. 2). Similar to LSM, communities were responsible for carrying out all HI activities, with HI committees providing community-level oversight of activities and monitoring progress. In this manuscript, ‘HI’ refers to the specific set of structural house improvements implemented in the MMP LSM/HI trial, as defined above.

### Costing approach

This is a retrospective cost analysis from a societal perspective using a combination of ‘top-down’ and ‘bottom-up’ approaches [30]. The societal perspective includes both provider (health system) and community costs and is considered the gold standard in economic evaluation. Top-down costing involves allocating programme level costs to component activities while bottom-up costing involves estimating resource use at a micro level and then summing up to estimate total programme level costs. The ‘ingredients approach’ which involves identifying quantities of inputs (ingredients) used and their unit costs was used. Analysis was conducted for financial and economic costs [31]. Financial costs are those where money changes hands, whereas economic costs also include non-financial (e.g. donated) resources in addition to financial costs; and thus accounts for all resources consumed [22, 31].

### Resource identification and valuation

Trial protocol and operating manuals were reviewed to populate a list of all activities conducted in each of the three intervention arms (i.e. the control arm was excluded), and the activities were categorized into pre-implementation and implementation phases. Activities were categorized as programmatic, i.e. necessary for routine, intervention implementation, based on field operating manuals. Research activities, defined as those not necessary for routine implementation, were excluded from the analysis. For each programmatic activity, requisite resources (‘ingredients’) and their quantities were identified from protocols, operating manuals, inventories, activity logbooks, progress reports and socio-economic sub-studies; and clarified with project staff [25, 28]. The proportion of full time employment (%FTE) was used to estimate staff time spent on trial implementation (as opposed to research or non-trial related activities) based on discussions with relevant project staff members; estimated staff costs (%FTE × salary) were then allocated to the relevant activity (Additional file 1). Programme management and overhead costs were directly allocated to trial arms according to estimated proportions of use [22]. Unit prices of purchased inputs were valued based on purchase prices extracted from financial records (excluding taxes). Donated resources, and purchased inputs for which unit prices were missing in financial records, were valued using market prices, e.g. local supplier price catalogues for physical items or services; or Malawian minimum wage rates (for non-skilled domestic labour) for community members’ time donation, assuming the Ministry of Health would pay communities in programmatic implementation.

All cost data was entered into a Microsoft Excel spreadsheet and summarized into cost categories, including staff, training, donated labour, consumables, transport, equipment and malaria workshops. Equipment with a value of more than US$100 and a useful life of more than 1 year were defined as capital costs and treated separately in the analysis (below). Thus the final spreadsheet captured quantities and unit costs of each resource input (purchased and donated) used in implementing LSM and HI in the MMP LSM/HI trial.

### Cost allocation to trial arms

**Fig. 3:**
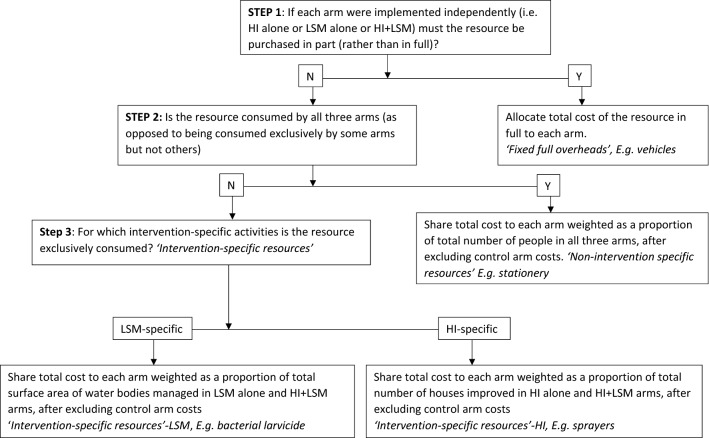
Algorithm showing decisions taken when sharing costs of shared resources to trial intervention arms. Due to the factorial design of the trial, all resources were shared between at least two trial arms

Given the factorial trial design, all resources were shared by at least two arms. Thus, suitable proxies were used to allocate the estimated total cost of each input to the respective consuming trial arms. Briefly, (1) purchase costs for capital items which would only be purchased in full assuming each arm were implemented independently, e.g. vehicles, were allocated in full to each arm; (2) HI-specific resources required for implementing HI-related activities, e.g. wire mesh, were allocated proportionately to HI-containing arms, i.e. HI only and HI + LSM arms, but not LSM only arm; (3) LSM-specific resources required for implementing LSM-related activities, e.g. larvicide, were proportionately allocated to LSM-containing arms, i.e. LSM only and HI + LSM arms, but not HI only arm; (4) non-intervention specific resources, e.g. stationery, were allocated proportionately to all arms. Proxies were weighted for the number of households for HI-related items; habitat size for LSM-related resources; and number of people per arm for non-intervention specific resources (Fig. 3, Additional file 2).

### Financial and economic cost analysis

#### Cost estimation

Resources were separated into financial (where money changed hands) and non-financial if donated, including pre-existing resources (e.g. project administration offices), community members’ time and materials. The financial costing included financial inputs only; while the economic costing considered both financial and non-financial costs. For all resource inputs, the original (consumption) currency, i.e. Malawi Kwacha (MWK), Euro or USD, and year was recorded for all costs. Costs were inflated to 2017 values and then converted to equivalent 2017 USD values using year-average International Financial Statistics inflation and exchange rates (https://data.imf.org/regular.aspx?key=61545862), as described in Turner et al*.* [32] (see Additional file 3). Capital item costs were annualized in the financial costing by dividing the purchase price by the useful life and similarly, with discounting at 3% in the economic costing, so that only the value of the capital item used during the project lifetime was included in the analysis. For each resource, unit cost was multiplied by quantity consumed to estimate the total financial or economic cost. For each intervention arm, costs were summed up for all activities/resources incurred. However, no further adjustments to costs for any potential effects of the interventions beyond the trial period were made, as explained in more detail below.

#### Timeframe of costing

The MMP LSM/HI trial included a 28-month pre-implementation preparatory phase and a 24-month trial implementation period. For average annual costs calculations (see “Cost indicators”), no adjustments were made for any lifespan of each intervention arm beyond that of the 2-year trial period, in contrast to, for example, the 3-year lifespan conventionally assumed when annualizing LLIN distribution costs. Specifically, the effects of the HI, LSM, or HI + LSM interventions were not regarded to last beyond the trial period, and thus total annual per person costs only reflect the observed trial period of 2 years. Additionally, because the pre-implementation phase included multiple preparation stages, from designing the malaria workshops and establishing the intensive community engagement program, to coordination with the national LLIN distribution campaign, the pre-implementation phase costs were excluded from annual per person cost calculations, as these pre-implementation phase costs were deemed unlikely to represent scale-up or programmatic implementation. However, as much as possible, pre-implementation costs reflect only those activities and time periods relevant to preparations for implementation of trial interventions, including community sensitization and trainings. Once-off activities carried out in the pre-implementation period only but considered core to the interventions were adjusted as though they occurred in the trial period and thus included in annual average cost calculations, e.g. brick making for HI [28].

#### Cost indicators

The total cost (financial or economic) of each intervention arm is aggregated as *total programme cost*; *total average annual cost*; and *per household and person per year costs*.

The *total programme cost* for each intervention, i.e. total cost from start to completion of the project, was the sum of allocated costs to that intervention. The *total average annual cos*t is the average of implementation years 1 and 2 costs, which excluded preparatory phase costs (as above). *Per household* and *per person* costs are the total average annual costs divided by the number of households in that intervention arm; and the total average annual cost divided by the number of people in the intervention arm, respectively. See summary box.

**Table:** 

Summary box: Cost indicators and recommended interpretation	
*Total programme cost* The cost incurred by the Majete Malaria Project (MMP) to implement larval source management (LSM) and house improvement (HI) in the 2 × 2 factorial cluster-randomized trial. This estimate includes both implementation and pre-implementation phase costs and is relevant for programmes/studies implementing LSM and house improvement (HI) in a factorial design, as in the MMP Average annual cost per household per year* the average annual cost incurred per household covered when implementing either LSM or HI or HI + LSM, as in the MMP trial Average annual cost per person per year* the average annual cost incurred per person covered when implementing either LSM or HI or HI + LSM, as in the MMP trial *These costs are based on the average of implementation years 1 and 2, and exclude pre-implementation phase costs. These costs were considered to be relevant for national control programmes as practical estimates for routine programmatic implementation	

To aid in planning scale-up, costs are also presented as pre-implementation and implementation phase; by major cost category; and capital and non-capital. Since the aim was to guide implementation scale-up, shared costs were allocated so that each intervention arm had the requisite resources (including administration and village-level resources) required to be implemented independently of the other intervention arms. Consequently, the grand sum of estimated total costs of implementing each arm as standalone exceeds total programme costs, but more accurately represents the costs of delivering the intervention arms individually.

For each intervention arm, results are presented in 2017 US$ for the total, annual, and per person and household financial and economic costs. Costs presented below are economic, unless otherwise specified. Financial and economic costs are presented in Tables 1, 2 and 3.

**Table 1:** Financial and economic implementation costs (2017 US$) of house improvement (HI) alone

Cost category	Pre-implementation phase^a^		Implementation costs (Y1)^a^		Average annual costs^a^		Percentage of total annual average	
	Financial	Economic	Financial	Economic	Financial	Economic	Annual financial	Annual economic
Recurrent costs								
Staff (international)	49,032	49,032	59,069	59,069	46,039	46,039	42.6	37.3
Staff (national)	20,078	20,078	18,354	18,354	14,268	14,268	13.2	11.6
Training	12,276	12,276	1840	1840	3738	3738	3.5	3.0
Transport^b^	5123	5123	14,890	14,890	12,399	12,399	11.5	10.0
Office consumables and supplies	–	–	882	882	836	836	0.8	0.7
Community labour (manual)^c^	–	–	–	7108	–	3554	0	2.9
Community time attending village workshops^d^		6323		7281		7564	0	6.1
IEC and other community engagement activities^e^	2361	2361	1767	1767	2200	2200	2.0	1.8
Office space and storage	–	3190	–	4224	–	3216	0.0	2.6
Communication	1926	3484	3138	4674	2321	3491	2.2	2.8
Capital costs^f^								
Transport^b^	24,911	24,911	24,731	24,731	24,580	24,580	22.8	19.9
Computers and accessories	1987	1987	154	154	77	77	0.1	0.1
Screening wire mesh and accessories	–	–	1563	1563	1540	1540	1.4	1.3
Sub-total recurrent costs	90,797	101,868	99,940	120,089	81,802	97,306	75.7	78.8
Sub-total capital costs	26,898	26,898	26,295	26,295	26,197	26,197	24.3	21.2
Total costs	117,695	128,766	126,388	146,537	107,999	123,503	100.0	100.0
Cost per person					23.64	27.04		

^a^Pre-implementation phase: Jan 2014–April 2016. Y1 = year 1 (May 2016–April 2017). Year 2 = May 2017–May 2018 (cost not shown). Implementation phase = year 1 and year 2. Annual average costs = (year 1 + year 2)/2

^b^Transport: Recurrent transport costs include vehicle operating costs only (fuel, insurance, maintenance and repairs). Capital transport costs include vehicle purchase costs only

^c^Manual labour donated by community members towards HI activities: closing eaves, gaps in walls, and fixing wire mesh, etc., valued using Malawi government minimum wage rate

^d^Community time attending village workshops (person-hours spent per workshop), valued using Malawi government minimum wage rate

^e^IEC = Information, Education and Communication, including costs of printing and translation of educational material, implementation guides/manuals, etc. Other community engagement activities include community members labour towards disseminating messages about interventions (e.g. village ‘criers’ to inform community members of upcoming meetings

^f^Capital costs: Costs for items with a useful life of > 1 year and replacement value of > $100

**Table 2:** Financial and economic implementation costs (2017 US$) of Larval Source Management (LSM) alone

Cost category	Pre-implementation phase^a^		Implementation costs (Y1)^a^		Average annual costs^a^		Percentage of total annual average	
	Financial	Economic	Financial	Economic	Financial	Economic	Annual financial	Annual economic
Recurrent costs								
Staff (international)	29,419	29,419	59,069	59,069	57,829	57,829	41.6	33.9
Staff (national)	12,047	12,047	18,370	18,370	17,910	17,910	12.9	10.5
Training	15,499	15,499	11,645	11,645	8919	8919	6.4	5.2
Transport^b^	5469	5469	15,643	15,643	13,996	13,996	10.1	8.2
Bacterial larvicide (*Bti*, CIF)^c^	–	–	5883	5883	6244	6244	4.5	3.7
Office consumables and sundries	–	–	1313	1313	1245	1245	0.9	0.7
Community labour (manual)^d^	–		–	12,492	–	13,085	0.0	7.7
Community time attending village workshops^e^		9393	–	12,212	–	12,679	0.0	7.4
IEC and other community engagement activities^f^	3074	3074	2631	2631	3275	3275	2.4	1.9
Office space and storage	–	1914	–	4224	–	4005	0.0	2.4
Communication	1156	2090	3138	4674	2858	4315	2.1	2.5
Small equipment^g^	–	–	850	850	474	474	0.3	0.3
Capital costs^h^								
Transport^b^	25,659	25,659	25,503	25,503	25,312	25,312	18.2	14.9
Computers and accessories	1987	1987	154	154	77	77	0.1	0.1
Personal protective equipment for spraying	–	–	974	974	1035	1035	0.7	0.6
Sub-total recurrent costs	66,664	78,906	118,542	149,006	112,750	143,976	81.0	84.5
Sub-total capital costs	27,646	27,646	26,630	26,630	26,423	26,423	19.0	15.5
Total costs	94,310	106,553	145,172	175,636	139,173	170,399	100	100
Cost per person					20.46	25.06		

^a^Pre-implementation phase: Jan 2014–April 2016. Y1 = year 1 (May 2016–April 2017). Year 2 = May 2017–May 2018 (cost not shown). Implementation phase = year 1 and year 2. Annual average costs = (year 1 + year 2)/2

^b^Transport: Recurrent transport costs include vehicle operating costs only (fuel, insurance, maintenance and repairs). Capital transport costs include vehicle purchase costs only

^c^Cost and insurance and freight for bacterial larvicide (*Bti*: *Bacillus thuringiensis israelensis*, AM625 strain, commercial name: VectoBac WDG [Valent Biosciences, Libertyville IL, USA]). Shipped from the USA to Malawi

^d^Manual labour donated by community members towards LSM activities: draining, filling and bacterial larvicide application to water bodies; mapping water bodies; and monitoring; etc. valued using Malawi government minimum wage rate

^e^Community time attending village workshops (person-hours spent per workshop), valued using Malawi government minimum wage rate

^f^IEC = Information, Education and Communication, including costs of printing and translation of educational material, implementation guides/manuals, etc. Other community engagement activities include community members labour towards disseminating messages about interventions (e.g. village ‘criers’ to inform community members of upcoming meetings

^g^Other small equipment not meeting threshold for “capital” items (e.g. buckets for drawing water, hoes, shovels donated from communities). See (h)

^h^Capital costs: Costs for items with a useful life of > 1 year and replacement value of > $100

**Table 3:** Financial and economic implementation costs (2017 US$) of house improvement and Larval Source Management combined

Cost category	Pre-implementation phase^a^		Implementation costs (Y1)^a^		Average annual costs^a^		Percentage of total annual average	
	Financial	Economic	Financial	Economic	Financial	Economic	Annual financial	Annual economic
Recurrent costs								
Staff (international)	49,032	49,032	59,069	59,069	57,829	57,829	45.3	39.3
Staff (national)	20,078	20,078	18,353	18,353	17,894	17,894	14.0	12.2
Training	11,863	11,863	4330	4330	4953	4953	3.9	3.4
Transport^b^	5097	5097	14,917	14,917	13,462	13,462	10.5	9.2
Bacterial larvicide (*Bti*, CIF)^c^	–	–	1573	1573	1669	1669	1.3	1.1
Office consumables and supplies	–	–	882	882	822	822	0.6	0.6
Community labour (manual)^d^	–	–	–	8383	–	6020	0.0	4.1
Community time attending village workshops^e^	–	6087		7669	–	7967	0.0	5.4
IEC and other community engagement activities^f^	2315	2315	1702	1702	2119	2119	1.7	1.4
Office space and storage	–	3190	–	4224	–	4005	0.0	2.7
Communication	1926	3484	3138	4674	2858	4315	2.2	2.9
Small equipment^g^ (e.g. buckets for drawing water)	–	–	227	227	127	127	0.1	0.1
Capital costs^h^								
Transport^b^	24,854	24,854	24,673	24,673	24,525	24,525	19.2	16.7
Computers and accessories	1987	1987	154	154	77	77	0.1	0.1
Screening materials and accessories	–	–	1370	1370	1369	1369	1.1	0.9
Sub-total recurrent costs	90,313	101,147	104,192	126,003	101,732	121,181	79.7	82.4
Sub-total capital costs	26,841	26,841	26,197	26,197	25,971	25,971	20.3	17.7
Total costs	117,154	127,989	130,388	152,200	127,704	147,152	100.00	100.00
Cost per person					29.02	33.44		

^a^Pre-implementation phase: Jan 2014–April 2016. Y1 = year 1 (May 2016–April 2017). Year 2 = May 2017–May 2018 (cost not shown). Implementation phase = year 1 and year 2. Annual average costs = (year 1 + year 2)/2

^b^Transport: Recurrent transport costs include vehicle operating costs only (fuel, insurance, maintenance and repairs). Capital transport costs include vehicle purchase costs only

^c^Cost and insurance and freight for bacterial larvicide (*Bti*: *Bacillus thuringiensis israelensis*, AM625 strain, commercial name: VectoBac WDG [Valent Biosciences, Libertyville IL, USA]). Shipped from the USA to Malawi

^d^Manual labour donated by community members towards HI and LSM activities: closing eaves, gaps in walls, and fixing wire mesh; draining, filling and bacterial larvicide application to water bodies; mapping water bodies; and monitoring;

^e^Community time attending village workshops (person-hours spent per workshop), valued using Malawi government minimum wage rate

^f^IEC = Information, Education and Communication, including costs of printing and translation of educational material, implementation guides/manuals, etc. Other community engagement activities include community members labour towards disseminating messages about interventions (e.g. village ‘criers’ to inform community members of upcoming meetings

^g^Other small equipment not meeting threshold for “capital” items (e.g. buckets for drawing water, hoes, shovels donated from communities). See (h)

^h^Capital costs: Costs for items with a useful life of > 1 year and replacement value of > $100

#### Sensitivity analysis

The robustness to and effect of structural assumptions and parameter uncertainty on estimated total and per person costs was explored in one- and multi-way probabilistic sensitivity analyses using @RISK software^®^ v 7.6 (Palisade Incorporated, USA); an add-in to Microsoft Excel. Cost categories were included in sensitivity analysis if they accounted for ≥ 25% of total annual average economic costs. A predetermined decision was made to explore the effect of changes in population covered as it is a key driver in cost per person calculations [31]. Thus staff costs and population covered were included in sensitivity analysis simulations; all other input categories were excluded (see Additional file 4). The cost per person for each arm when values of staff costs and population covered were varied was simulated (100,000 iterations per simulation), as follows. For each iteration, input values were randomly sampled from a triangular distribution (defined by minimum, most likely, maximum values) of possible values of staff costs and population covered to calculate the total/per person costs, holding other input costs constant. For population covered, −/+ 20% changes (as minimum and maximum values, respectively) from the trial baseline mean estimates were assumed. For staff costs, the minimum was defined as total staff costs when all salaries were paid using local salary scales without changing the staff structure; i.e. international staff paid as nationals. The trial estimate was taken as maximum value. It was assumed that staff salaries in a trial represent highest possible personnel costs compared to routine implementation where government programme staff are usually used. For both staff and population covered, the trial mean estimates were taken as the ‘most likely’ value. For each simulation, the cost per person was summarized as a frequency distribution (summarized as mean, 5th and 95th percentile limits) of the 100,000 iteration estimates. Microsoft Excel^®^ 2016 was used for all data management and analysis.

## Results

### Overview

In the MMP, both house improvement (‘HI’) and larval source management (LSM) were implemented over a 28-month pre-implementation preparatory phase and a 24-month implementation phase, i.e. the “trial period” (as described in “Methods”). The period under evaluation thus spans January 2014 to May 2018; the pre-implementation period (up to 30th April 2016) and the trial period (through to 31st May 2018). The total programme cost of implementing HI, LSM and HI + LSM (pre-implementation phase through to implementation years 1 and 2) was estimated at $626,152.

### Housing improvement

House improvement only arm was implemented in 13 villages, covering 4568 people from 1030 households. The total average annual cost was $123,503. The average cost per household per year was $119.91 while the cost per person was $27.04 (Table 1).

Staff costs were the main cost driver, accounting for 48.9% of annual average implementation costs. Transport costs were the second major cost driver: 29.9% of annual average costs. Screening material and related equipment (i.e. wire mesh, nails and scissors) represented < 2% of annual average economic costs. Estimated donated time from communities was estimated at $22,235 in the implementation phase, and represented 9% of annual average costs (Table 1). Implementation year 1 accounted for 59% of total implementation phase costs. Overall, non-capital costs accounted for 79% of annual average costs.

### Larval source management

Larval source management only was implemented in 24 villages, covering 6801 people, across 1520 households. The total average annual cost of implementing LSM was estimated as $170,399. The average cost per household per year was $112.10 while the cost per person was $25.06 (Table 2). Staff costs were the main cost driver, accounting for 44.4% of annual average implementation costs. Transport costs were the second major cost driver: 23.1% of annual average implementation costs. Bacterial larvicide costs (cost, insurance and freight) represented 3.7% of annual average implementation costs. Estimated donated time from communities was valued at $51,528, representing 15.1% of annual average implementation costs. Time spent attending village workshops represented half of estimated community costs. Overall, non-capital costs accounted for 85% of annual average costs in the implementation phase, being similarly distributed between years 1 and 2 (52 vs 48%, respectively) (Table 2).

### House improvement plus larval source management

House improvement plus larval source management arm was implemented in nine villages, covering 4400 people across 952 households. The total average annual cost of implementing HI + LSM was estimated as $147,152. The average cost per household per year was $154.57 while the cost per person per year was $33.44. Staff salary costs were the main cost driver, accounting for 51.5% of annual average implementation costs. Bacterial larvicide and wire mesh represented 2% of annual average implementation costs. Transport costs accounted for 26% of annual average costs in the implementation phase. Estimated donated time from communities was valued at $27,973, and represented 9.5% of annual average implementation costs; of which malaria village workshops represented two-thirds. Overall, non-capital costs represented 82% of annual average implementation costs, which were similarly distributed across implementation years 1 and 2 (Table 3).

### Sensitivity analysis

In one-way sensitivity analysis, estimated annual average total and per person costs for all intervention arms were sensitive to staff costs and population covered. Assuming that all project staff were paid as nationals (i.e. no expatriate staff) while maintaining the same staff structure, and maintaining all other variables, reduced the average annual total and per person cost for each intervention arm by at least $38,000 and $8, respectively, i.e. > 34% change in both total and per person costs.

**Fig. 4:**
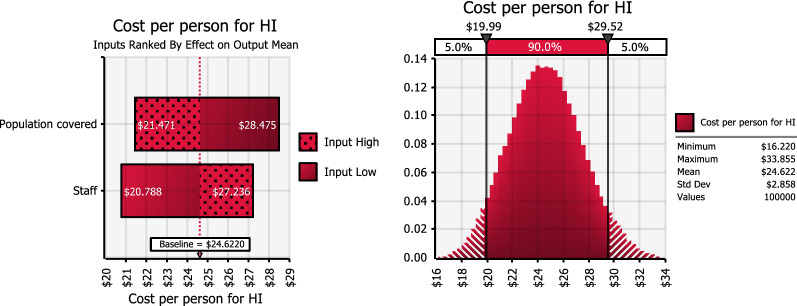
Tornado graph of sensitivity analysis for mean cost per person for house improvement alone arm. The simulated effect of changes in staff costs and population covered on estimated mean cost per person. The left panel shows the change in cost per person from the baseline, i.e. trial mean estimate, when staff costs and population covered are varied. The lighter (dotted) shade of the bar corresponds to increasing input values. The darker (solid fill) shade corresponds to decreasing input values. For both LSM and HI + LSM, the cost per person increases with increasing staff costs; and reduces with increasing population covered; and vice versa. The right panels show the corresponding frequency distribution of simulated estimates of cost per person. The area bound by the black vertical line represents the upper limits of the 5th and 95th percentiles (i.e. 90% uncertainty interval) of simulated cost per person estimates of HI: US$19.99–29.52. The minimum, most likely and maximum values, respectively, used for the distributions were: Staff cost: $ 43,798; $ 120,616; $ 120,616. Population covered: 3654; 4568; 5482

Including staff costs and population covered together in a multi-way sensitivity analysis resulted in an estimated cost per person between $15–$34 for all interventions (see Fig. 4 for HI arm tornado graph, and Additional file 4 for LSM and HI + LSM tornado graphs).

## Discussion

This study estimated the incremental cost of community-led HI and LSM, when implemented alone or in combination, in addition to standard NMCP strategies, within a cluster-randomized trial. The incremental per person costs of implementing HI, LSM or HI + LSM, i.e. $27.04, $25.06 and $33.44, respectively, in 2017 US$, from a societal perspective were high compared to programmes of varied design reported elsewhere. In Kenya and Tanzania, three larviciding-only programmes cost between US$ 1.14 and US$ 3.09 per person per year (author-adjusted to 2017 US$) [14]. Rahman et al. reported ten-fold lower than costs estimated in this study: US$ 1.23 per person per year (PPPY) (author-adjusted to 2017 US$) for a seasonal larviciding-only programme delivered over 2 years in Tanzania [33]. Notably, both studies did not report on draining or filling, and adopted a provider perspective, i.e. excluded community costs.

In the Gambia, a provider-led house screening intervention cost $11.34 PPPY (adjusted to USD 2017), and could have increased to $12.63 PPPY if communities purchased locally available wire mesh, as opposed to free-of-charge donation [19].

Caution is encouraged when comparing findings from this study with the above cited studies for two main reasons. Firstly, these findings are absolute costs and thus comparisons with other studies where cost-effectiveness is unknown may be misleading. Whereas previous studies often reported ‘cost per person *protected*’, i.e. interventions protected (or were assumed to protect) the target population [14, 15, 33], this study used ‘cost per person’ as there was no statistical evidence of a protective effect of HI or LSM on primary entomological and epidemiological outcomes [24]. Thus, these annual per person costs are best interpreted as the average annual cost of implementing the interventions, as in the Majete trial study area. Secondly, differences in the design and components of interventions likely influenced differences in costs. In this study, larval source management included draining and filling, whereas previous studies were predominantly larviciding-only, although the term ‘larval source management’ was used. Consequently, the resource requirements will differ depending on which of draining, filling or larviciding are included. For example, while proportions of water bodies treated with larvicide may be similar across settings, e.g. 86% in the rural Tanzania study [33] versus 84% in the MMP LSM/HI trial [24], also see Additional file 5), the costs of draining and filling, not reported in the Tanzania study, may underlie observed differences. Similarly for house improvement interventions, previous studies have reported closing eaves and screening windows, but varied in terms of additional modifications, e.g. door way modifications, thus costs, and effectiveness on epidemiological outcomes [18, 20], may differ. Moreover, even where both windows and eaves are screened, material used for screening/closing may differ, e.g. metal wire mesh with/without polyvinyl chloride coating. In this study, metal wire mesh was only used for screening windows/ventilations spaces, and mud bricks for closing eaves and doorway modifications. Other studies used cement and fire-heated bricks [19, 21], thus costs will differ. For both LSM and house improvement, the optimal combination of components to include for sustainable programmatic implementation, e.g. draining/filling/larviciding for LSM; and closing eaves/screening windows/door modifications for house improvement, is not always clear and requires further investigation [20, 34].

Similarly, lack of consistency in operational indicators, i.e. units of houses ‘improved’ or larval sources ‘managed’, compared to e.g. LLINs, i.e. ‘number of nets delivered’ or ‘number of people sleeping under a bed net’, makes comparison of costs (and cost-effectiveness) challenging for LSM and house improvement studies and programmes. The development and adoption of consistent operational indicators for LSM and house improvement could improve comparability with other interventions and better guide funders and policy makers.

Other reasons that could explain the high costs observed in this study include: the all year-round implementation compared to spatial- or temporal-targeting; relatively high (international) staff costs; low population density in the trial area; and/or the considerable community involvement, which included a community engagement programme in addition to implementation of LSM and HI. Moreover, the higher economic (compared to financial) costs in this study are due to donated labour costs, reflecting the community-led approach to implementation; and donated office and storage space.

In the MMP LSM/HI trial, interventions were designed to be implemented all year-round. For LSM, the timing and duration of implementation is an important determinant of costs [13]. In the MMP, bacterial larviciding was intended to be repeated weekly, throughout the year. Consequently, bacterial larvicide and application labour costs are high. In Tanzania and Kenya, larviciding was temporally targeted to coincide with the rainy season [14, 33]. For house improvement, the timing of the intervention may not significantly impact the estimated cost given that house improvement activities (closing eaves and fixing wire mesh) were a one-off activity, assuming that community time donation was valued equally throughout the year as was the case in this study. For both HI and LSM, community members attended malaria village workshops at least fortnightly, which increased the estimated economic costs over and above the financial costs as community members were not paid for attending these workshops. Similarly, health animators (including village committee members) spent additional time in community mobilization activities and intervention-specific planning, monitoring and evaluation activities. However, where providers want to compensate village workshop attendees for their time, the financial costs would be expected to be higher than estimated in this study.

In addition, staff costs were estimated to reflect actual involvement in intervention implementation in the trial (using %FTE). This decision affected both number and type of staff (national and international) included in the analysis. Similar studies have included international staff, albeit to varying degrees. While the LSM/HI interventions in this study were designed as a community-led intervention, it will likely be the case that technical support and coordination from outside the communities will be required to ensure consistency in quality and provide monitoring and evaluation support [13]. In this study, this was provided by international research staff, but it is envisaged that as capacity to support LSM and house improvement increases in Malawi, as seen in other countries across Africa, that these interventions could increasingly be supported with in-country expertise. This is a critical step towards long-term sustainability. The effect of excluding some or all of the international experts in the present study on the fidelity of the implementation process is unclear. Consequently, excluding staff based only on cost savings may not be appropriate. Further research is needed to elucidate how best to sustainably implement community-led interventions. Moreover, even for national staff, there might be efficiency gains in routine implementation settings where existing staff assume more responsibilities, reducing project staff costs. This was not explicitly explored in this analysis. Notwithstanding, staff salaries were adjusted for %FTE time contributions so that, as much as possible, costs reflect actual involvement in implementation activities; however, this was imprecise owing to the complex factorial design and possible recall bias when estimating %FTE.

The study area was a rural setting. For LSM, the choice of urban versus rural setting is particularly important for two reasons. Firstly, with fewer built structures and different land use practices (e.g. more agricultural land use), more and/or larger water bodies suitable for malaria vector larvae may need to be treated in rural villages than urban areas [13]. Consequently, especially for bacterial larvicide and associated application labour costs, the total cost of LSM is likely to be higher in rural versus urban areas, holding other factors constant. Secondly, lower human population densities often associated with rural compared to urban areas, result in smaller denominators in cost per capita calculations; even where size and/or number of water bodies are similar because LSM targets breeding sites in a geographical area rather than the human population per se, compared to ITN programme costs, which should change proportionately with changes in human population [13]. The population density in the MMP catchment area was 96 people/km^2^ reflecting the rural nature of the study area (compared to 3334 people/km^2^ for Blantyre City in 2018) [23, 35]. Though similar to a rural area in Tanzania (47.1 people/km^2^), population densities in previous seasonal, larviciding-only programmes were much higher: 1082/km^2^ in rural Kenyan highlands [33]. In the 1930s, a successful environmental management programme that included larval source management and house screening implemented over a 20-year period in the Zambian Copperbelt covered an average 664 people/km^2^ [12]. Nonetheless, while per person costs should decrease with increasing human population density covered, the exact form of this relationship is not clear, and may not necessarily be simple linear as it is also affected by the human population-to-larval habitat density ratio [13]. Transport costs were also high, mainly because of the geographical clustering of study villages (‘focal areas’). Distances covered between study villages/clusters and field site/project management office to deliver commodities and for coordination and sensitization meetings were substantial and likely contributed to higher costs (23–34% of total costs) (see Additional file 6).

For HI, relatively fewer built dwelling structures usually associated with rural areas should imply fewer houses to improve, hence lower intervention costs. However, the low population density results in smaller denominators in per capita costs (e.g. 1982 houses covered; on average 4.5 people/household in this study), compared to where few houses are occupied by a large number of people, i.e. large denominators, hence low per capita costs. Moreover, even where there are few houses to improve, the impact of typically large share of programme management costs, may not be completely offset by a low population covered [36].

Finally, the community-led approach adopted by MMP, compared to predominantly provider-led approaches in other studies, is noteworthy. The benefits of community involvement in the design of malaria interventions have been reviewed elsewhere. To eliminate malaria, communities must take ownership of control interventions [37–39]. This was the rationale for MMP’s approach, using existing THP community engagement infrastructure. However, placing the ultimate responsibility of implementing interventions on communities could have impacted costs estimated in this study in at least two ways. First, the financial costing does not capture community opportunity costs, thus estimated financial costs are lower than economic costs. Secondly, the value placed on communities’ labour is likely lower than would be the cost of (semi-) skilled personnel specifically trained in and tasked with LSM and HI activities, hence the estimated cost in this study (using government minimum wage) is lower than would be in the latter scenario. Alternative valuations of communities’ donated time, e.g. using skilled personnel wage rates should increase the estimated costs. Necessarily, the present study adopted a societal perspective, in contrast to the cited studies from Kenya, Tanzania and The Gambia where the provider perspective was used [22]; although communities may perceive their opportunity costs to be higher than estimated in this study, which has implications for sustainability. Nevertheless, estimated donated community costs might have been overestimated: *Bti* application and malaria village workshops, for example, were planned to be conducted every 1 and 2 weeks, respectively. However, some villages conducted fewer applications and/or meetings, hence actual costs could have been lower [25]. Furthermore, the costs of pre-existing community engagement infrastructure was included as THP’s staff time. However, where such community engagement structures (e.g. the epicentre approach) need to be established first, implementation costs for similar community-led approaches may be higher than reported in this study.

The main strength of this study is the presentation of costs by arm, which could guide planners of similar or modified designs in future (cost-) effectiveness studies or routine implementation programmes. Furthermore, this study has reported the first cost analysis of a community-led house improvement intervention. For the Malawi NMCP, the scenario with all staff paid as nationals could be used as a practical budgeting cost estimate for routine implementation of HI, LSM and HI + LSM. Cost savings are possible as efficiency and capacity utilization (e.g. for transport, staff costs, trainings) are maximized, alongside possible increasing economies of scale. Intervention design, e.g. larviciding duration for LSM, also presents opportunities for cost savings, although this should be informed by effectiveness data. Where existing control programme/ministry of health infrastructures (including offices, community engagement processes, monitoring and evaluation support) can be leveraged, implementation costs may be lower than in this study, although community labour may still be substantial. Such integration may enhance sustainability, as long as community’s education and perceptions of benefit are sustained.

However, this cost analysis has several methodological limitations. Due to the factorial design, geographical clustering of interventions and management structure of the trial, cost allocation of shared resources to individual intervention arms was based on proxies rather than precise resource use; therefore, the estimated costs may have been imprecise. Furthermore, this study did not fully consider economies of scale. It is possible that cost per person would be lower where interventions are implemented at large scale (i.e. increasing surface area and people covered), however evidence from scale up of ITN programmes shows that economies of scale occur only at very large programme size, and for IRS programmes, economies of scale are not consistently achieved [40, 41]. Similarly, this study did not quantify and explore the effect of resource capacity utilization changes, e.g. personnel, vehicles, buildings. It is possible there might be cost savings with increasing capacity utilization and efficiency of included inputs [36]. The retrospective data collection approach rendered quantity estimates prone to recall bias, i.e. in project staff interviews.

Furthermore, while capital items were annualized by only including the value of the resource consumed during the project implementation, any potential effect of the interventions beyond the trial period was not explored. For LSM, the effect of *Bti* lasts 7 days, thus justifying weekly application; and draining/filling may need to be repeated within a calendar year. House improvements should last longer, but previous studies on structural house improvements did not assess integrity beyond 2 years [21, 42]. Notwithstanding the decision to limit the life span of the interventions to the trial period, the results are presented by year to allow the effect of sustained life spans on these interventions beyond the 2-year period, which may determine sustainability, to be explored. Moreover, possible wider societal benefits not measured in the MMP trial period, e.g. jobs and socioeconomic developments associated with funding for LSM [43] or house improvements, may encourage community uptake and sustainability. Importantly, any possible impact on residual transmission potentially resulting from sustained house improvements when LLIN use declines due to very low risk of infection, may make house improvements more favorable. In the MMP LSM/HI trial areas, LLIN ownership and use was high immediately after the national distribution campaign but declined steadily within 2 years [24]. In contrast, house improvements could offer protection to people not using LLINs.

The probabilistic sensitivity analysis in this study was limited in several ways. As parameter estimates were not necessarily sampled, and in the absence of published cost estimates, arbitrarily determined percentage changes and triangular distributions were used. This renders the analyses prone to several criticisms. First, the effect of arbitrary percentage changes on parameter estimates is likely to be predictable, driven by magnitude of percentage change, and may not be informative. Therefore, the utility of uncertainty in other important cost categories which would have otherwise been explored, e.g. fixed percentage changes to staff %FTE, was limited. Second, the changes tested may not necessarily be realistic or likely [22, 36]. Furthermore, the effect of using different proxies in sensitivity analyses was not explored, although these were very important in this study. The proxies used were the authors’ best efforts at allocating shared costs in a complex trial, compared to e.g. allocating costs equally across intervention arms which, although explored, the approach was deemed to be methodologically poor and uninformative. Therefore, shared costs were replicated across trial arms, assuming that each arm would be implemented independently, as in Mangham-Jefferies et al. [44], although this decision may have overestimated total costs. For economic evaluations alongside factorial trials, there is no consensus how to allocate and analyse shared costs, and how costs (and effects) interact, which ultimately compromises comparability between studies [45].

Finally, while cost-effectiveness is more informative for resource allocation where multiple options are available, and was originally planned, it was not conducted as there was no statistical evidence that the interventions improved epidemiological or entomological outcomes [24]. Nevertheless, cost analysis is useful for planning interventions in other settings where effectiveness may be different.

## Conclusions

The costs of implementing community-led HI and LSM, alone or in combination, as implemented in the MMP LSM/HI trial were comparable. The estimated cost of implementing each arm can inform future implementation of some or all of the studied interventions. Moreover, since delivering packages of combination interventions is associated with increasing economies of scope, the scenarios and approach presented in this study may be useful for costing future trial and control programme designs; however, the importance of any proxies used will need to be carefully explored. Compared to previous studies, a societal perspective was required to capture the full range of costs of community-led implementation of interventions. This decision may limit comparability. However, as community involvement is important for community ownership and sustainability, future economic evaluations of similarly designed studies or programmes should consider adopting the societal perspective, as in this study. Future economic evaluations of LSM and house improvement should include cost-effectiveness outcomes to facilitate comparisons of costs with other malaria control interventions; cost-effectiveness is also more intuitive for funders and policy-makers [22, 31].

## Supplementary Information

**Additional file 1.** Staff structure, roles and time spent on intervention implementation of trial interventions.

**Additional file 2.** Resource consumption shares for each trial intervention arm used to develop cost allocation proxies.

**Additional file 3. ** Inflation, exchange and annualization factors.

**Additional file 4.** Tornado graphs and sensitivity analysis of the mean cost per person for larval source management alone arm and house improvement plus larval source management arm.

**Additional file 5.** Summary of actions taken by communities to manage water bodies in the Majete Malaria Project larval source management and house improvement trial. *Bti*: *Bacillus thuringiensis israelensis*, AM625 strain, commercial name: VectoBac WDG [Valent Biosciences, Libertyville IL, USA]). Source: McCann et al. unpublished data.

**Additional file 6. **Map of the Majete Malaria Project larval source management and house improvement trial catchment area showing the relative locations of trial villages, organized in groups (red boundaries), called ‘Focal areas’ (FA) A, B and C) in which the interventions were implemented, project field offices (located in Chikwawa district) and the program manager’s office, located in Blantyre City. Base Maps adapted from Google Maps. The main road network connecting the project manager’s (COM) office and field sites is shown in yellow and white.

## Data Availability

The spreadsheet used and analysed during the current study are available from the authors upon reasonable request.

## Abbreviations

WHO: World Health Organization
ITN: Insecticide-treated net
LSM: Larval source management
LLIN: Long-lasting insecticidal nets
HI: House screening
IRS: Indoor residual spraying
ACT: Artemisinin-based combination therapy
RDTs: Rapid diagnostic tests
IPTp: Intermittent preventive therapy in pregnancy
NMCP: National Malaria Control Programme
MMP: Majete Malaria Project
