# Supplementary material for: Cost of community-led larval source management and house improvement for malaria control: a cost analysis within a cluster-randomized trial in a rural district in Malawi
Source: Malar J. 2021 Jun 13;20:268. doi: 10.1186/s12936-021-03800-4 (PMC8200285; doi:10.1186/s12936-021-03800-4)
Supplement: Supplementary file 1 — Additional file 1. Staff structure, roles and time spent on intervention implementation of trial interventions. [file 12936_2021_3800_MOESM1_ESM.docx]

Table S1.1: Staff structure, roles and estimated proportion of staff time spent on implementation of house improvement (HI) and larval source management (LSM) in the Majete Malaria Project (MMP) HI/LSM trial

| **Institution ^a^** | **Position** | **Roles and responsiblities (implementation activities, research exluded)** | **% Average FTE ^b^** |
| --- | --- | --- | --- |
| CoM | Program manager | Overall progam management and support to technical field team. Oversight on procurement of equipment and consumables | 50% |
| CoM | Program assistant | Support the program manager with management and support, especially procurement | 50% |
| WUR | Team leader - Entomologist | Responsible for overall project execution of interventions in the communities | 50% |
| WUR | Community engagement specialist | Responsble for development of training and implementation manuals  Designing and planning health animator/HAS/training-of-trainer trainings | 25% |
| CoM | Malariologist-CoM-NMCP liason | Provide liason with National Malaria Control Programme, on behalf of MMP | 10% |
| CoM | Drivers | Transporting MMP staff and health animators to/from villages, training and coordination meetings, etc.  Transporting supplies e.g. Bti and wire gauze to communities | 70% |
| CoM | Site Coordinator | Assisting Team Leader in day-to-day management of execution of interventions in communities, inlcuding coordinating logistics and supply of intervention-related equipment; overseeing trainings; communication with communities; liasing with program manager's office | 50% |
| CoM | Field Supervisor | Assisting Site supervisor with coordinating logistics and supply of intervention-related equipment; overseeing trainings; communication with communities; liasing with program manager's office | 70% |
| CoM | Entomologist | Technical supervisor for implementation and monitoring and evaluation of malaria workshops, HI and LSM | 30 |
| CoM | Field technicians | Technical day-to-day assistance in implementation activities , and through coordination meetings  Assisting with distribution of supplie to communities | 70% |
| THP | THP Extension Project Officers | Managing the health animators, the committees, and all of their activities. | 40% |
| DHO | Health surveillance assistants | Provided expert knowledge on malaria topics during | 10% |

^a^ Institutions: CoM: University of Malawi College of Medicine, WUR: Wageningen University Research; THP: The Hunger Project; DHO: District Health Office, Government of Malawi.

^b^ %FTE: proportion of full time employment spent on implementation activities. FTE differed by year and accounted for in the costing analysis. Average shown for conciseness.

NMCP: National Malaria Control Programme
