# Supplementary material for: Cost of community-led larval source management and house improvement for malaria control: a cost analysis within a cluster-randomized trial in a rural district in Malawi
Source: Malar J. 2021 Jun 13;20:268. doi: 10.1186/s12936-021-03800-4 (PMC8200285; doi:10.1186/s12936-021-03800-4)
Supplement: Supplementary file 2 — Additional file 2. Resource consumption shares for each trial intervention arm used to develop cost allocation proxies. [file 12936_2021_3800_MOESM2_ESM.docx]

**Supplementary File 2: Estimates of resource input share for House Improvement (HI) and Larval Source Management (LSM) used for developing proxies for allocating shared costs between trial arms. Estimates are derived from intervention implementation surveys**

Table S2.1: HI arms (only reported for villages allocated to implement HI)

| Trial arm | Number of villages | Number of houses sampled | Number of houses completed* per arm | Proportion of houses completed* |
| --- | --- | --- | --- | --- |
| HI | 13 | 1584 | 933 | 0.58495298 |
| HI+LSM | 9 | 1154 | 662 | 0.41504702 |
| Total | 22 | 2738 | 1595 | 1 |

* Houses considered completed when windows and ventilation gaps within wall were covered with wire mesh and eaves were closed.

Table S2.2: LSM arms (only reported for villages allocated to implement LSM)

| Trial arm | Total number of villages | Total number of habitats | Total habitat area (m^2^) | Proportion of number of habitats per arm | Proportion of habitat area |
| --- | --- | --- | --- | --- | --- |
| LSM | 24 | 323 | 143537.5767 | 0.58941606 | 0.789068275 |
| HI+LSM | 9 | 225 | 38370.1 | 0.41058394 | 0.210931725 |
| Total | 33 | 548 | 181907.6767 | 1 | 1 |

Table S2.3: Trial population covered – used for sharing non-intervention-specific shared costs to all three trial arms

| Trial arm | Total number of people | Proportion out of total number of people in trial area | Number of households sampled | Proportion of total number of households sampled |
| --- | --- | --- | --- | --- |
| HI | 4568 | 0.289682288 | 1030 | 0.29411765 |
| LSM | 6801 | 0.431289238 | 1520 | 0.43403769 |
| HI+LSM | 4400 | 0.279028474 | 952 | 0.27184466 |
| Total | 15769 | 1 | 3502 | 1 |
