# Supplementary material for: Cost of community-led larval source management and house improvement for malaria control: a cost analysis within a cluster-randomized trial in a rural district in Malawi
Source: Malar J. 2021 Jun 13;20:268. doi: 10.1186/s12936-021-03800-4 (PMC8200285; doi:10.1186/s12936-021-03800-4)
Supplement: Supplementary file 3 — Additional file 3. Inflation, exchange and annualization factors. [file 12936_2021_3800_MOESM3_ESM.docx]

**Inflation and deflation factors, and exchanges rates**

**Table S3.1: Inflation and deflation factors for Malawi Kwacha**

| **Year *** | **Price** | **Inflator** | **Deflator** |
| --- | --- | --- | --- |
| 2013 | 166.12 |  |  |
| 2014** | 205.65 | 1.237961 | 0.820565 |
| 2015 | 250.62 | 1.218673 | 0.821624 |
| 2016*** | 305.03 | 1.217102 | 0.896514 |
| 2017 **** | 340.24 | 1.115431 | 0.889516 |
| 2018***** | 382.5 | 1.124206 | - |

Source: International Financial Statistics, Consumer Price Index, All items. (Accessed 3 July 2019) <https://data.imf.org/regular.aspx?key=61545861>

* 2010=100 units

** Start of pre-implementation phase

*** Start of implementation phase

**** Base case year in which costs were reported

***** Final year of implementation phase. Some costs made in first half of 2018

**Table S3.2: Inflation and deflation factors for Euro**

| Year * | **Price** | **Inflator** | **Deflator** |
| --- | --- | --- | --- |
| 2013 | 107.4827 |  |  |
| 2014** | 108.5318 | 1.00976 | 0.994033 |
| 2015 | 109.1832 | 1.006002 | 0.996843 |
| 2016*** | 109.529 | 1.003167 | 0.986374 |
| 2017 **** | 111.0421 | 1.013815 | 0.98325 |
| 2018***** | 112.9337 | 1.017035 | - |

Source: International Financial Statistics, Consumer Price Index, All items. (Accessed 3 July 2019) <https://data.imf.org/regular.aspx?key=61545861>

* 2010=100 units

** Start of pre-implementation phase

*** Start of implementation phase

**** Base case year in which costs were reported

***** Final year of implementation phase. Some costs made in first half of 2018

**Table S3.3: Exchange rates**

| **Year** | **US $ value** | **€ equivalent** | **MWK equivalent** |
| --- | --- | --- | --- |
| 2013 | 1.00 | 0.73 | 434.96 |
| 2014 | 1.00 | 0.82 | 470.78 |
| 2015 | 1.00 | 0.92 | 672.68 |
| 2016 | 1.00 | 0.95 | 728.62 |
| 2017 | 1.00 | 0.83 | 732.03 |

Source: International Financial Statistics, National Currency per U.S. Dollar, End of period. (Accessed 3 July 2019) <https://data.imf.org/regular.aspx?key=61545862>

**Table S3.4: Annualisation factors**

| **Useful life (in years)** | **Annualisation factor (at 3% discount rate)** |
| --- | --- |
| 2 | 1.91 |
| 3 | 2.83 |
| 4 | 3.72 |
| 5 | 4.58 |
| 6 | 5.42 |

Source: Phillips, Mills, Dye. Guidelines for Cost-effectiveness anaylsis of vector control. 1993. WHO
