# Supplementary material for: Cost of community-led larval source management and house improvement for malaria control: a cost analysis within a cluster-randomized trial in a rural district in Malawi
Source: Malar J. 2021 Jun 13;20:268. doi: 10.1186/s12936-021-03800-4 (PMC8200285; doi:10.1186/s12936-021-03800-4)
Supplement: Supplementary file 4 — Additional file 4. Tornado graphs and sensitivity analysis of the mean cost per person for larval source management alone arm and house improvement plus larval source management arm. [file 12936_2021_3800_MOESM4_ESM.docx]

A

B

C

D

Figure S4.1: **The simulated effect of changes in staff costs and population covered on estimated mean cost per person of implementing larval source management (LSM) alone (A and B) and house improvement and LSM combination (C and D)**. The vertical axes shows input variables; the output (cost per person) is shown on the horizontal axes.

**Interpretation:** The left panels (A and C) show the change in cost per person from the baseline, i.e. trial mean estimate, when staff costs and population covered are varied. The lighter (dotted) shade of the bar corresponds to increasing input values. The darker (solid fill) shade corresponds to decreasing input values. For both LSM and HI+LSM, the cost per person increases with increasing staff costs; and reduces with increasing population covered, and vice versa. The right panels (C and D) show the corresponding frequency distribution of simulated estimates of cost per person. The area bound by the black vertical line represents the upper limits of the 5th and 95th percentiles (i.e. 90% uncertainty interval) of simulated cost per person estimates of LSM and HI+LSM: US$18.81-27.27; and US$24.28-36.28, respectively.

For LSM alone (A and B): the minimum, most likely and maximum values used for the triangular distributions in the simulation were:

Staff costs: US$ 54,988; $ 151,477; $ 151,477; population covered: 5441; 6801; 8161

For HI and LSM combination (C and D): the minimum, most likely and maximum values used for the triangular distributions in the simulation were:

Staff cost: US$ 54,955; US$ 151,444; US$ 151,444; population covered: 3520; 4400; 5280

Table S4.1: Rationale for excluding and including resource input categories in sensitivity analysis

| **Input category** | **Variation tested** | **Rationale for exclusion/inclusion in sensitivity analysis** |
| --- | --- | --- |
| Staff | Salary scales. All staff assumed to be paid at local salary rates, i.e. no expatriate rates | Staff accounted for a large proportion of estimated total costs. Total costs dependent on staff structure, number, salary rates (i.e. national or expat rates). Both factors represent possible variations where other implementation approaches use different staffing structures and salary scales. Salary scales only were varied. Staff structure was not varied as effect on fidelity of interventions unclear |
| Training | Not tested | Training cost were determined by nature/content to be delivered to make the intervention delivery possible. Therefore, alterations were deemed likely to change fidelity of intervention, and therefore not tested |
| Transport | Not tested | Point estimates derived from best efforts to capture distance required to be covered to deliver intervention to communities. Total cost based on distance covered x fuel price. Distance therefore not be varied, otherwise trips may not be fulfilled. Changes in fuel prices assumed to be taken into account in inflation adjustments |
| Bti/larvicide | Not tested | No major assumptions in calculation of input costs. Fluctuations in unit prices unlikely to significantly impact costs, as small contribution to mean trial total costs estimates |
| Other consumables | Not tested | No major assumptions in calculation of input costs. Fluctuations in unit prices unlikely to significantly impact costs, as small contribution to mean trial total costs estimates |
| Community time attending village workshops | Not tested | Time attending village workshops represents communities' time, estimated as person-hours. Normal changes in minimum wage expected but assumed to be taken care of in inflation adjustment which should cover changes in wages over time |
| Community manual labour | Not tested | Manual labour represents communities' time, estimated as person-hours. Normal changes in minimum wage expected but assumed to be taken care of in inflation adjustment which should cover changes in wages over time |
| Community Engagement and IEC | Not tested | Composite of critical activities for communities to adopt interventions. Changes in quantities could impact quality of implementation |
| Office space and storage | Not tested | No basis to change office space requirements as size management and field teams would be affected by the change in space available. Rental costs based on local rates and deemed appropriate reference |
| Communication | Not tested | No major assumptions in calculation of input costs. Fluctuations in unit prices unlikely to significantly impact costs, as small contribution to mean trial total costs estimates |
| Computers and accessories | Not tested | No major assumptions in calculation of input costs. Fluctuations in unit prices unlikely to significantly impact costs, as small contribution to mean trial total costs estimates |
| Other small equipment | Not tested | No major assumptions in calculation of input costs. Fluctuations in unit prices unlikely to significantly impact costs, as small contribution to mean trial total costs estimates |
| Wire mesh | Not tested | No major assumptions in calculation of input costs. Fluctuations in unit prices unlikely to significantly impact costs, as small contribution to mean trial total costs estimates |
| Population covered | Number of people covered under each intervention arm  +/- 20% from trial estimates | Estimates were from a census conducted in 2015. Migrations, births and deaths could have led to changes in population covered and could affects per capita costs. Extreme percentage changes were chosen to explore the effect of implementing interventions in more densely populated areas on per capita costs. |
