# Supplementary figures and images for: Cost of community-led larval source management and house improvement for malaria control: a cost analysis within a cluster-randomized trial in a rural district in Malawi
Source: Malar J. 2021 Jun 13;20:268. doi: 10.1186/s12936-021-03800-4 (PMC8200285; doi:10.1186/s12936-021-03800-4)

# Action taken by LSM committee

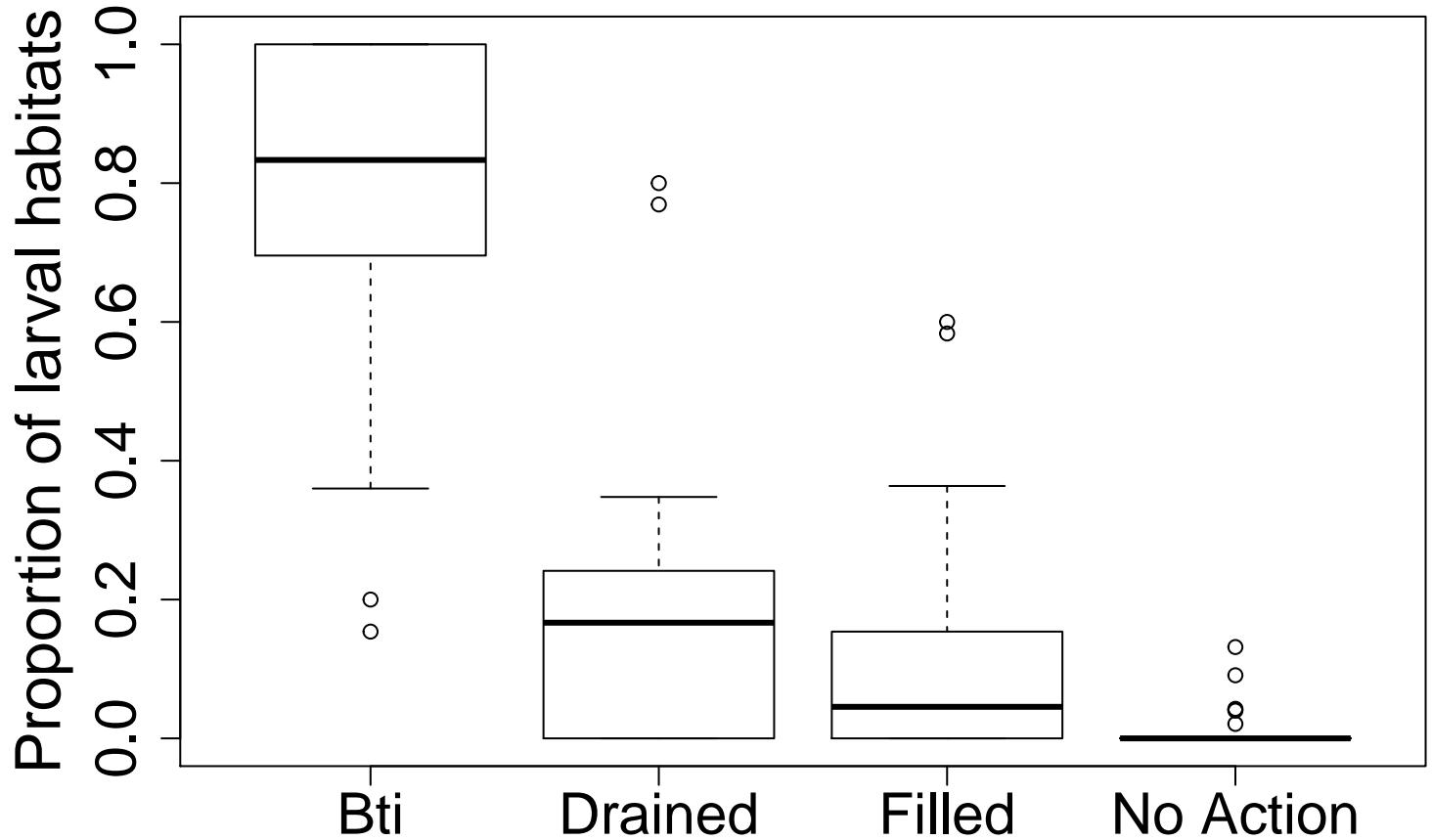

Supplement: Supplementary file 5 — Additional file 5. Summary of actions taken by communities to manage water bodies in the Majete Malaria Project larval source management and house improvement trial. Bti: Bacillus thuringiensis israelensis, AM625 strain, commercial name: VectoBac WDG [Valent Biosciences, Libertyville IL, USA]). Source: McCann et al. unpublished data. [file 12936_2021_3800_MOESM5_ESM.pdf]

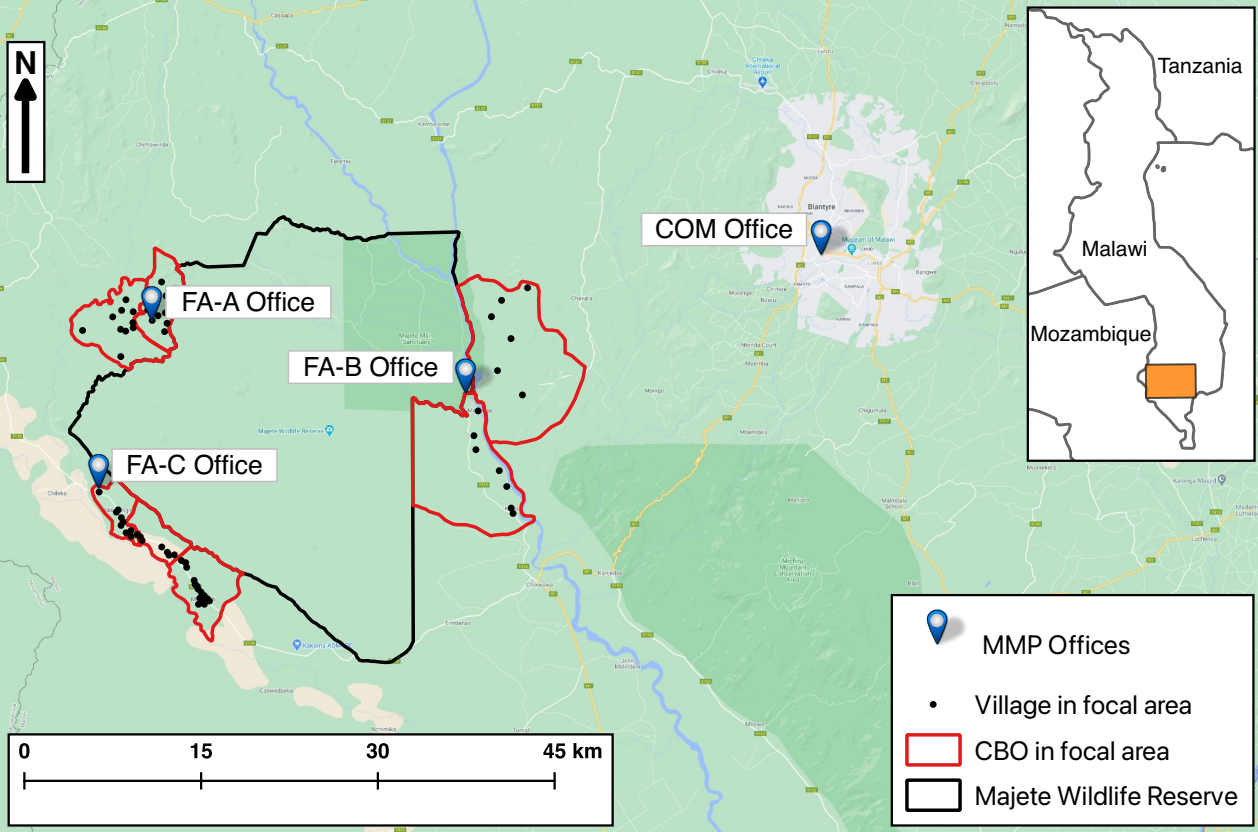

Supplement: Supplementary file 6 — Additional file 6. Map of the Majete Malaria Project larval source management and house improvement trial catchment area showing the relative locations of trial villages, organized in groups (red boundaries), called ‘Focal areas’ (FA) A, B and C) in which the interventions were implemented, project field offices (located in Chikwawa district) and the program manager’s office, located in Blantyre City. Base Maps adapted from Google Maps. The main road network connecting the project manager’s (COM) office and field sites is shown in yellow and white. [file 12936_2021_3800_MOESM6_ESM.pdf]
